# Vascular mimicry induced by m^6^A mediated IGFL2-AS1/AR axis contributes to pazopanib resistance in clear cell renal cell carcinoma

**DOI:** 10.1038/s41420-023-01423-z

**Published:** 2023-04-11

**Authors:** Bo Cheng, Mingyue Xie, Yong Zhou, Tian Li, Wanting Liu, Wenjing Yu, Man Jia, Shuang Yu, Lixuan Chen, Rongyang Dai, Ronghao Wang

**Affiliations:** 1 Department of Urology, the Affiliated Hospital of Southwest Medical University, Luzhou, 646000 China; 2 Department of Biochemistry and Molecular Biology, School of Basic Medical Sciences, Southwest Medical University, Luzhou, 646000 China

**Keywords:** Cancer therapeutic resistance, Nuclear receptors

## Abstract

Metastatic clear cell renal cell carcinoma (ccRCC) is a lethal sub-type of kidney cancer. Vascular mimicry (VM) has been postulated as an alternative route to supply tumors with nutrients, playing key role in tumor development. Whether VM development is linked to pazopanib efficacy, however, remains unclear. Here, our in vitro and in vivo models identified that VM development was profoundly increased in pazopanib resistant ccRCC as compared to the sensitive controls, which was due to the activation of IGFL2-AS1/AR/TWIST1 signaling. IGFL2-AS1, a m^6^A modified long coding RNA, was demethylated by METTL3/METTL14 complex and stabilized owing to its failing recognition by YTHDF2 upon chronic pazopanib treatment. Further mechanistic dissection illustrated that IGFL2-AS1 physically interacted with the 5’-UTR AR mRNA and neutralized the negative regulation of 5’-uORF (upstream open reading frame) on AR translation. Indeed, IGFL2-AS1 short of AR binding region failed to promote AR expression, VM formation and pazopanib resistance. In vivo xenografted mouse model also elucidated that inhibition of AR activity with enzalutamide or silence of IGFL2-AS1 with siRNAs all led to retarded growth of pazopanib resistant ccRCC tumors. Together, these results suggest that IGFL2-AS1 may represent a key player to mediate pazopanib-induced VM formation of ccRCC cells via regulating AR expression and targeting this newly identified IGFL2-AS1/AR signaling may help us to better suppress ccRCC VM formation and to increase the therapeutic efficacy of pazopanib.

## Introduction

Kidney cancer is the 14th most common cancer worldwide and clear cell renal cell carcinoma (ccRCC) takes up approximate 80% cases [1]. The most recent estimates suggest that approximate 79,000 cases of kidney cancer will be newly diagnosed and near 14,000 deaths will be reported in United States during 2022. Even worse, some ccRCC will progress to metastatic disease, which remains a main cause of ccRCC related death and has a under 10% 5-year survival rate [2, 3]. Understanding of the pathogenesis and progression of this disease at the molecular level has facilitated the development of tyrosine kinase inhibitor (TKI) or immunotherapy to treat metastatic ccRCC [4–6]. Although immunotherapy alone or combined with TKI is a popular way to treat metastatic ccRCC [6, 7], TKI such as pazopanib is still utilized as the first line therapy to treat metastatic ccRCC in developing countries, showing promising outcomes [8–11]. Pazopanib suppresses tumor neo-angiogenesis by blocking the activities of platelet-derived growth factor receptor (PDGFR), vascular endothelial growth factor receptor (VEGFR) and fibroblast growth factor receptor (FGFR) on the surface of endothelial cells or malignant cells [9]. However, the effective duration of pazopanib therapy is only around 12 months and drug resistance is inevitably emerged. Until now, the mechanism underlying pazopanib resistance is still elusive and deserves deep investigation.

Endothelial mediated angiogenesis plays a critical role in connecting the tumor micro-environment and cancer cells via the constantly secreted chemokines or cell adhesion molecules [12, 13], and has been viewed as the only way for blood supply. Previous studies have demonstrated that immunosuppression mediated by tumor-associated macrophages (TAMs) or tumor associated fibroblasts (TAFs) are highly related to anti-angiogenetic resistance [12, 14, 15]. However, the direct effect of anti-angiogenetic drugs on cancer cells has been ignored. The aim of this study is to investigate how ccRCC cells respond to pazopanib treatment at the molecular level.

Vasculogenic mimicry (VM) has been discovered in 1999 as new route to provide nutrients and blood for tumor independent of angiogenesis [16]. It refers to a transformation of malignant tumor cells into endothelial-like cells, forming tumor-derived vascular networks to provide blood or nutrients for tumor progression [17]. Thus, CD31 or CD34 negative and PAS positive vascular structure is widely viewed as VM [18]. In clinic, VM has been documented in various cancers including ccRCC and is positively associated with tumor malignancy, metastasis and shorter overall survival [19–21]. We hypothesize that ccRCC cells develop VM to escape pazopanib treatment and to facilitate drug resistance. A recent study has demonstrated that androgen receptor (AR) promotes ccRCC VM formation via enhancing TWIST1 expression [22]. However, whether AR-mediated VM is casually linked to pazopanib resistance of ccRCC cells and how AR is mechanistically regulated upon pazopanib treatment have not been investigated yet.

Here, our study is the first one establishing the linkage between VM and pazopanib resistance, identifying m^6^A mediated IGFL2-AS1/AR axis as one critical signaling pathway controlling this process. The results suggest that targeting IGFL2-AS1/AR axis may decrease VM number and improve pazopanib efficacy to suppress ccRCC progression.

## Results

### AR mediated VM formation is one mechanism driving ccRCC resistance to pazopanib

**Fig. 1:**
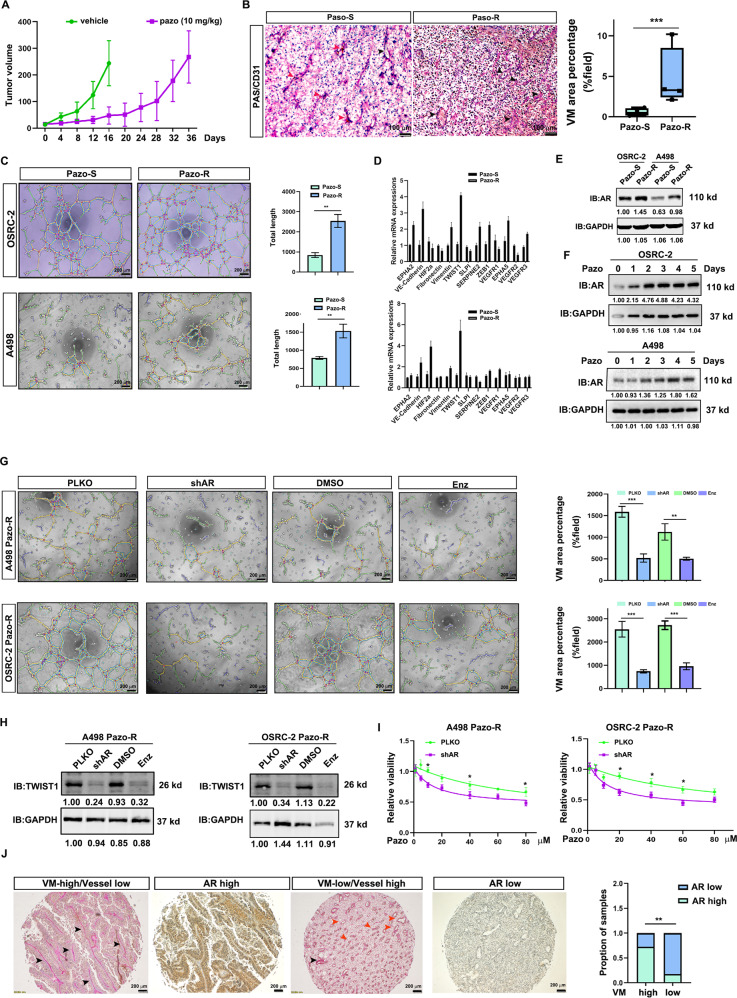
AR mediated VM formation is one mechanism driving ccRCC resistance to pazopanib. **A** Tumor growth curve of pazopanib or vehicle-administrated mice. 10 mg/kg pazopanib was intraperitoneally injected every other day. **B** CD31/PAS staining of pazopanib or vehicle-treated tumor section. Left, representative images of CD31/PAS staining. Right, a statistical analysis. Scale bar: 100 μm. Red arrow: vessels; Black arrow: vascular mimicry of tumor cells. **C** VM formation assays of OSRC-2 and A498 cells before and after pazopanib resistance. Left, representative images. Right, statistical analyses. Scale bar: 200 μm. **D** QPCR assays revealed the expression pattern of VM related genes in OSRC-2 and A498 cells before and after pazopanib resistance. Gene expression level was normalized to GAPDH mRNA. **E** Western blotting showed the expression level of AR in OSRC-2 and A498 cells before and after pazopanib resistance. GAPDH was loading control. **F** Transient pazopanib treatment also boosted AR expression in both OSRC-2 and A498 cells. GAPDH served as internal control. **G** AR inhibition with shRNAs or enzalutamide suppressed VM development of OSRC-2 and A498 pazopanib resistant cells. Left, representative images. Right, statistical analyses. Scale bar: 200 μm. **H** AR inhibition with shRNAs or enzalutamide decreased TWIST1 expression in OSRC-2 and A498 pazopanib resistant cells. GAPDH was used as loading control. **I** Knockdown of AR re-sensitized OSRC-2 and A498 pazopanib resistant cells to pazopanib treatment. **J** High AR ccRCC preferred to have higher VM number than low AR counterparts. Scale bar: 200 μm. Pazo=pazopanib, S Sensitive, R Resistant; *P < 0.05; **P < 0.01; ***P < 0.001.

Development of VM appear to be one essential route for primary tumors to supply cancer cells with nutrients. Although pazopanib has been utilized as one standard therapy to suppress the metastatic RCC, its impact on the VM development, however, remains unclear. To end this, we administrated OSRC-2 tumor with 10.0 mg/kg pazopanib every other day for 5 weeks to develop pazopanib resistant ccRCC tumor (Fig. 1A, *n* = 7). CD31/PAS staining elucidated that VM number was greatly increased in pazopanib-resistant ccRCC tumors, along with this was the reduced number of blood vessels (Fig. 1B). To verify this in vivo observation in vitro and to find the underlying molecular mechanism, we established two pazopanib resistant cell lines by continuously exposing them with pazopanib treatment (Supplementary Fig. 1A, B). In line with the in vivo finding, both OSRC-2 and A498 pazopanib-resistant cells harbored stronger VM forming ability as compared to their corresponding sensitive cells (Fig. 1C), which was confirmed by the upregulation of VM-related genes in pazopanib-resistant cells (Fig. 1D).

The significance of AR in ccRCC development including VM formation [22–27] prompted us to examine whether AR was involved in this process. As shown in Fig. 1E, A498 and OSRC-2 pazopanib resistant cells also expressed more AR than the corresponding controls. Transient pazopanib treatment also promoted AR expression in both OSRC-2 and A498 cells in time-dependent manner (Fig. 1F). To confirm whether AR was the casual factor determining VM formation and pazopanib resistance, we applied shAR or enzalutamide (clinically used as anti-androgen) to block AR signaling, which significantly suppressed VM formation and TWIST1 expression of OSRC-2 and A498 pazopanib resistant cells (Fig. 1G, H). Significantly, AR inhibition by shRNAs also increased pazopanib efficacy to suppress these two resistant cell lines (Fig. 1I), suggesting AR and it mediated VM at least partially contributed to ccRCC pazopanib resistant development. The connection between AR and VM was further clinically observed in ccRCC tissue microarray, which revealed that high AR was frequently detected in ccRCC patients with high VM development (Fig. 1J). Of note, pazopanib resistant OSRC-2 and A498 cells had stronger invasive ability l (Supplementary Fig. 1C) than the sensitive controls, which was coincided with the strong correlation between VM and tumor metastasis.

Together, results from Fig. 1A-J suggest that chronic pazopanib treatment increases AR expression, which promotes ccRCC VM formation and causes drug resistance.

### Determination of IGFL2-AS1 as the upstream regulator of AR in pazopanib resistant ccRCC cells

**Fig. 2:**
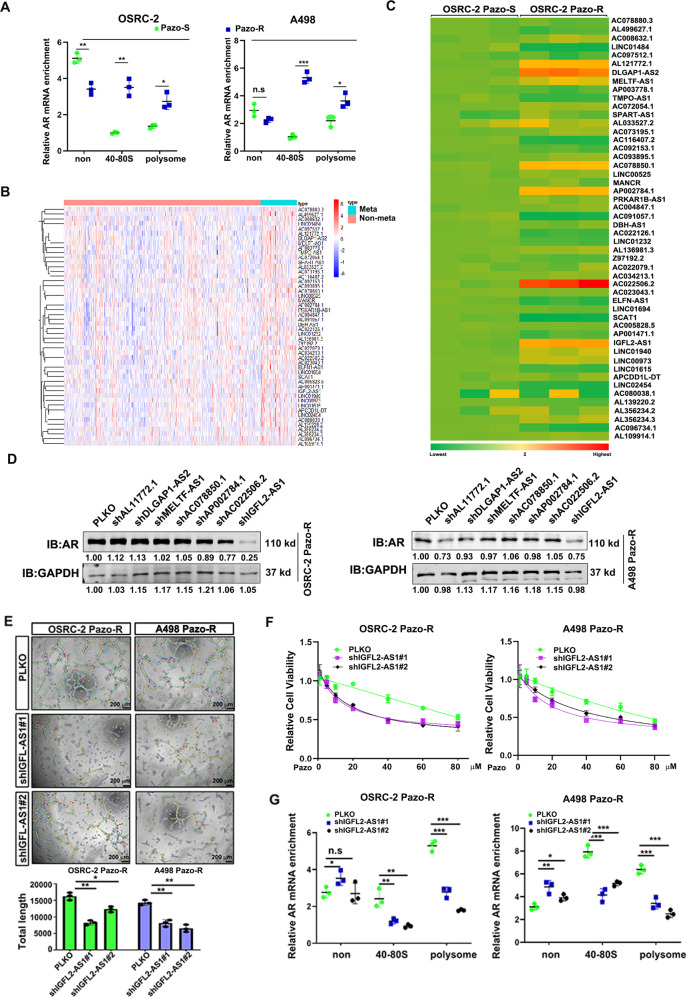
Determination of IGFL2-AS1 as the upstream regulator of AR in pazopanib resistant ccRCC cells. **A** Enrichment of AR mRNA with ribosomes in A498 and OSRC-2 before and after pazopanib resistance by polysome profiling assay and followed qPCR detection. **B** A heatmap of the top 50 lncRNAs highly expressed in metastatic ccRCC. **C** QPCR assay to detect the expression levels of top 50 metastatic lncRNAs in OSRC-2 pazopanib sensitive and resistant cells. Gene expression level was normalized to GAPDH mRNA. **D** AR expression level in OSRC-2 (left) and A498 (right) pazopanib resistant cells when lncRNA candidates was knocked down by shRNAs. GAPDH was loading control. **E** Knockdown of IGFL2-AS1 suppressed the VM development of OSRC-2 and A498 pazopanib resistant cells. Left, representative images. Right, statistical analyses. Scale bar: 200 μm. **F** Knockdown of IGFL2-AS1 restored sensitivity of OSRC-2 and A498 pazopanib resistant cells. **G** Knockdown of IGFL2-AS1 reduced the enrichment of AR mRNA with ribosomes. *P < 0.05; **P < 0.01; ***P < 0.001, n.s. no significance.

To unfold the underlying mechanisms responsible for pazopanib-induced AR expression, we first examined whether AR was transcriptionally regulated upon chronic pazopanib treatment. As shown in Supplementary Fig. 2A, AR mRNA was comparable between pazopanib-resistant cells and sensitive controls, suggesting no transcriptional regulation on *AR* gene under such circumstance. Moreover, both AR protein stability and mRNA stability displayed similar curves upon drug treatments between pazopanib-resistant cells and parental cells (Supplementary Fig. 2B, C). Interestingly, AR mRNA was highly enriched with ribosomes in pazopanib-resistant cells according to the polysome profiling assay (Fig. 2A), suggesting AR induction by pazopanib was attributable to its more efficient translation.

Given the facts that long non-coding RNAs (lncRNAs) have been documented as critical regulators to affect gene translation and VM formation has been frequently observed in metastatic tumors, we therefore focused on top 50 metastatic related lncRNAs in TCGA-KIRC (Fig. 2B). Among them, 7/50 lncRNAs were experimentally verified to be over-expressed in OSRC-2 pazopanib resistant cells as compared to sensitive controls (Fig. 2C). However, only knockdown of IGFL2-AS1 remarkably decreased AR protein level in both A498 and OSRC-2 pazopanib resistant cells (Fig. 2D). IGFL2-AS1 reduction also suppressed VM formation and restored sensitivity of pazopanib resistant A498 and OSRC-2 cells (Fig. 2E, F). More importantly, polysome profiling assay further confirmed that IGFL2-AS1 knockdown significantly reduced AR enriched level with ribosomes (Fig. 2G), suggesting IGFL2-AS1 indeed enhanced AR mRNA translation, leading to AR upregulation and VM formation.

Collectively, results from Fig. 2A–G suggest that IGFL2-AS1 is determined as the upstream regulator of AR to promote ccRCC VM and pazopanib resistance.

### IGFL2-AS1 regulates the uORF (upstream open reading frame) activity of AR mRNA and facilitates its translation

**Fig. 3:**
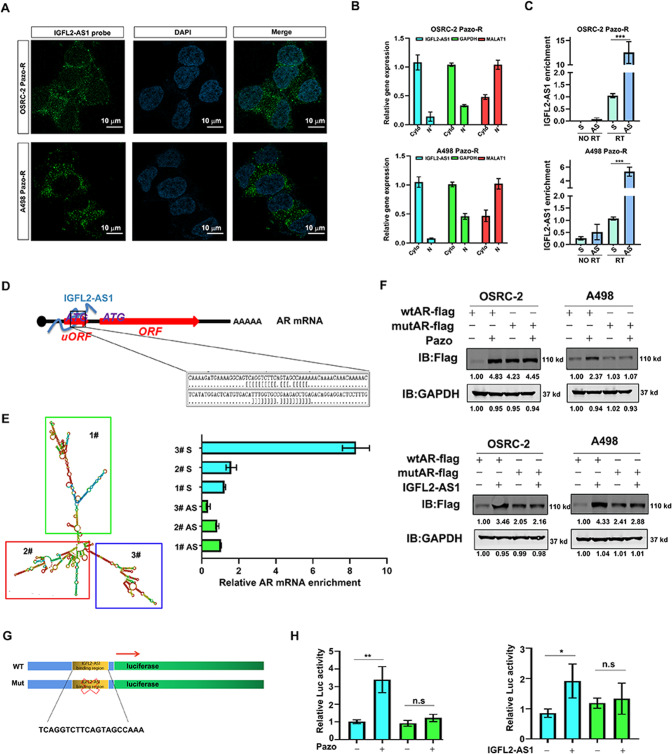
IGFL2-AS1 regulates the uORF activity of AR mRNA and facilitates its translation. **A** *FISH* staining of IGFL2-AS1 in pazopanib resistant ccRCC cells. **B** Cyto-nuclear fraction separation to identify IGFL2-AS1 location. Cytoplasmic RNA: GAPDH; Nuclear RNA: *MALAT1*. **C** RNA IP with 4 anti-sense probes against AR mRNA to detect IGFL2-AS1 enrichment. AS Anti-sense probe; S Sense probe. **D** Cartoon showing the interaction of IGFL2-AS1 and AR mRNA. **E** Left, RNA structure of IGFL2-AS1 predicted by *RNAfold Webserver*. Right, truncated mapping showed that fragment 3# containing AR binding motif interacted with AR mRNA. **F** Top, pazopanib treatment increased the expression of AR with wild type 5’-UTR but not that with ATG deficient 5’-UTR in both OSRC-2 and A498 cells. IGFL2-AS1 boosted the expression of AR with wild type 5’-UTR but not that with ATG deficient 5’-UTR in both OSRC-2 and A498 cells. GAPDH was internal control. **G** Cartoon showing how wild type and mutated AR 5’-UTR bearing luciferase constructs were made. **H** Pazopanib and IGFL2-AS1 enhanced the activity of wild type AR 5’-UTR bearing luciferase but not that of mutated one in OSRC-2 cells. *P < 0.05; **P < 0.01; ***P < 0.001, n.s = no significance.

IGFL2-AS1, 1.6 kb in length, has a low coding potential analyzed by Coding Potential Assessment Tool (Supplementary Fig. 3A, B). RNA fluorescence in situ *hybridization* (FISH) and cyto-nuclear separation all revealed that IGFL2-AS1 was mainly located in the cytoplasm in ccRCC cells (Fig. 3A, B).

Since translation occurs on mRNA, we sought to investigate whether IGFL2-AS1 could directly interact with AR mRNA and facilitate its translation. For this end, we used 4 biotin-labeled anti-sense probes of AR mRNA to pull down AR mRNA complex and noticed IGFL2-AS1 was profoundly precipitated in pazopanib resistant ccRCC cells (Fig. 3C), suggesting According to RNA-RNA prediction sofware (http://rtools.cbrc.jp/cgi-bin/RNARNA/index.pl), IGFL2-AS1 interacts with the 5’-UTR region of AR mRNA (Supplementary Fig. 3C, D). To confirm this binding, we synthesized biotin-labeled IGFL2-AS1 fragments according to the RNA structure predicted by *RNAfold Webserver* (Fig. 3E, left). RNA IP suggested that IGFL2-AS1 fragment 3# which contains the predicted binding RNA sequence interacted with AR (Fig. 3E, right) mRNA. Since AR mRNA 5’-UTR contains an upstream open reading frame (uORF), which potentially inhibits the downstream coding gene translation according to previous report. We hypothesized that IGFL2-AS1 binding interrupted the uORF translation, thus favoring the translation of AR coding region. To test it, we generated AR construct with either wild type uORF or mutated uORF in which the translation initiation code ATG was deficient. As exhibited in Fig. 3F, AR with mutated uORF (mutAR-flag) expressed abundant protein compared to that with wild type uORF (wtAR-flag). Concurrently, wtAR-flag but not mutAR-flag responded nicely to pazopanib and IGFL2-AS1 treatments (Fig. 3F).

To further confirm the biological functions of this AR mRNA uORF, we constructed wild type AR mRNA 5’-UTR as well as its mutant which lacks the IGFL2-AS1 binding region into the upstream of luciferase cDNA (Fig. 3G). As expected, both pazopanib and IGFL2-AS1 could increase the the activity of wild type AR 5’-UTR coupled luciferase (Fig. 3H). However, pazopanib and IGFL2-AS1 induced luciferase activity was lost in mutated construct (Fig. 3H), suggesting IGFL2-AS1 binding was indispensable for AR protein increase.

Together, results from Fig. 3A–H indicate that IGFL2-AS1 binds the 5’-UTR of AR mRNA and inhibits the activity of uORF, enhancing the downstream ORF translation.

### The contributions of IGFL2-AS1 to ccRCC VM formation and pazopanib resistance requires its binding to AR mRNA

**Fig. 4:**
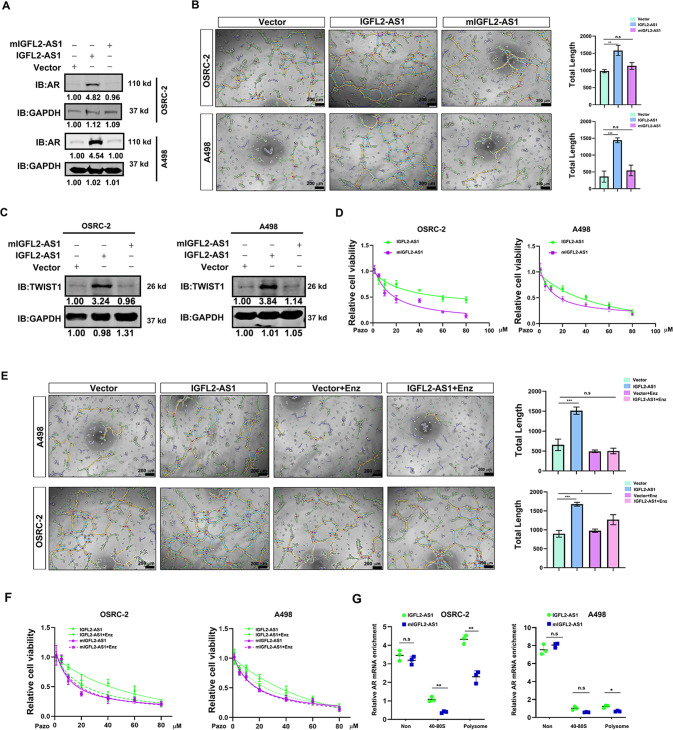
The contributions of IGFL2-AS1 to ccRCC VM formation and pazopanib resistance requires its binding to AR mRNA. **A** Wild type IGFL2-AS1 but not mutated one increased AR expression in A498 and OSRC-2 cells. GAPDH was normalizing control. **B** Wild type IGFL2-AS1 but not mutated one increased VM development of A498 and OSRC-2 cells. Left, representative images. Right, statistical analyses. Scale bar: 200 μm. **C** Wild type IGFL2-AS1 but not mutated one increased TWIST1 expression in A498 and OSRC-2 cells. GAPDH was loading control. **D** Wild type IGFL2-AS1 had stronger ability to promote pazopanib resistance of A498 and OSRC-2 cells than the mutated one. **E** Enzalutamide treatment attenuated IGFL2-AS1 induced VM development in A498 and OSRC-2 cells. Left, representative images. Right, statistical analyses. Scale bar: 200 μm. **F** Enzalutamide treatment abolished IGFL2-AS1 induced pazopanib resistance of ccRCC cells. **G** Wild type IGFL2-AS1 had stronger ability to promote AR translation than mutated one, monitored by polysome profiling assay. *P < 0.05; **P < 0.01; ***P < 0.001, n.s = no significance.

To confirm the functionality of IGFL2-AS1 requiring its binding to AR mRNA, we constructed ccRCC cell lines stably expressing wild type IGFL2-AS1 or mutated IGFL2-AS1 (mIGFL2-AS1) deficient of AR mRNA binding motif. As expected, IGFL2-AS1 but not mIGFL2-AS1 increased AR expression in both A498 and OSRC-2 cells (Fig. 4A). In addition, IGFL2-AS1 had stronger capacity to promote VM formation than the mutant one (Fig. 4B). Along with this, the expression level of TWIST1 was also increasingly regulated by wild type IGFL2-AS1 but not mIGFL2-AS1 (Fig. 4C). Significantly, A498 and OSRC-2 cells with IGFL2-AS1 introduction harbored increased pazopanib resistance as compared to the cells expressing mIGFL2-AS1 (Fig. 4D). To validate the impact of IGFL2-AS1 on ccRCC was AR dependent, we tested whether anti-androgen drug enzalutamide could impair IGFL2-AS1 induced VM formation and pazopanib resistance. As shown in Fig. 4E, F, IGFL2-AS1 induced VM formation and pazopanib resistance in A498 and OSRC-2 cells were profoundly attenuated by enzalutamide treatment. Significantly, an increased enrichment of AR mRNA with ribosomes was clearly examined in ccRCC cells expressing IGFL2-AS1 as compared to cells expressing mIGFL2-AS1 (Fig. 4G), strengthening the notion that IGFL2-AS1 interacted with AR mRNA and facilitated its translation.

Taken together, all these finding illustrate that the biological effects of IGFL2-AS1 on ccRCC VM and pazopanib resistance is dependent of its association with AR mRNA.

### IGFL2-AS1 m^6^A was demethylated and stabilized by YTHDF2 release upon pazopanib treatment

**Fig. 5:**
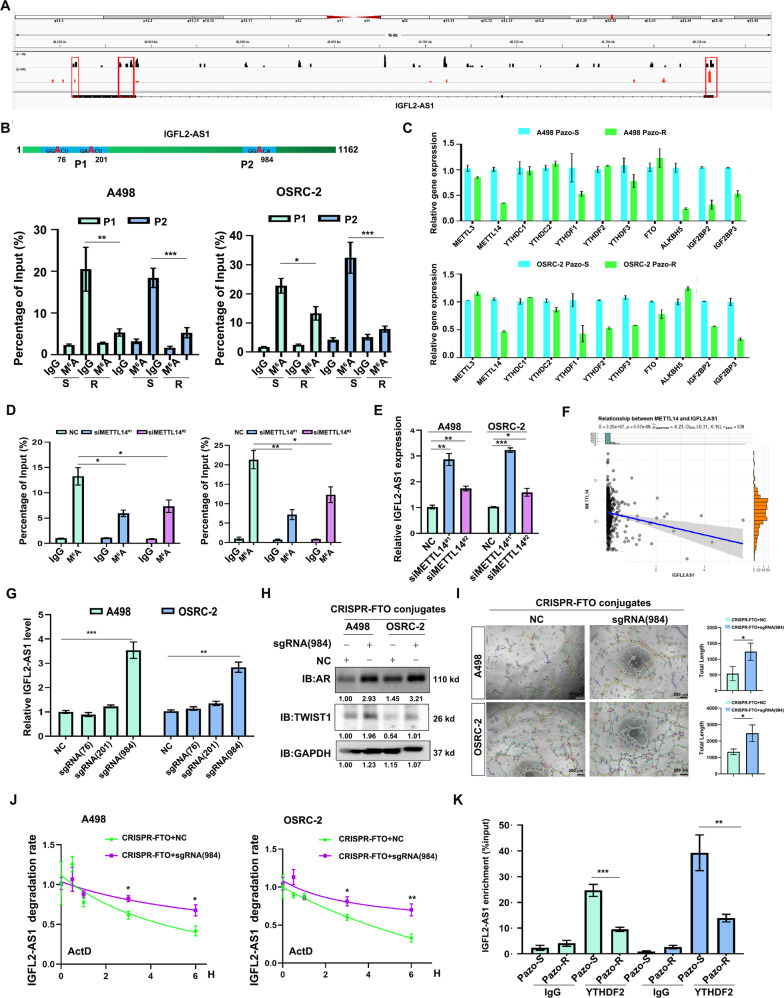
IGFL2-AS1 m^6^A was demethylated and stabilized by YTHDF2 release upon pazopanib treatment. **A** Analysis on m^6^A peaks of IGFL2-AS1 according to the GSE161304 miCLIP dataset. Red box indicated the m^6^A peaks. **B** Top, very high confidence of m^6^A sites as predicted by SRAMP. Bottom, m^6^A RIP revealed the differential m^6^A levels of IGFL2-AS1 between pazopanib sensitive and resistant ccRCC cells. P1, P2 were pairs of primers detecting the indicated region of IGFL2-AS1. **C** The mRNA expression levels of m^6^A regulators between pazopanib sensitive and resistant ccRCC cells. RNA level was normalized to GAPDH mRNA. **D** Knockdown of METTL14 with two independent siRNAs reduced m^6^A levels of IGFL2-AS1 in both A498 and OSRC-2 cells. **E** Knockdown of METTL14 with two independent siRNAs increased the expression level of IGFL2-AS1 in both A498 and OSRC-2 cells. GAPDH mRNA served as internal control. **F** METTL14 was negatively correlated with IGFL2-AS1 in TCGA-KIRC. **G** dCas9 CRISPR-FTO conjugates with sgRNA targeting A984 adjacent area of IGFL2-AS1 significantly increased its expression in both A498 and OSRC-2 cells. RNA level was normalized to GAPDH mRNA. **H** dCas9 CRISPR-FTO conjugates with sgRNA targeting A984 adjacent area of IGFL2-AS1 enhanced the expression levels of AR and TWIST1 in both A498 and OSRC-2 cells. GAPDH was loading control. **I** dCas9 CRISPR-FTO conjugates with sgRNA targeting A984 adjacent area of IGFL2-AS1 promoted VM development of A498 and OSRC-2 cells. Left, representative VM images. Right, statistical analyses of VM. Scale bar: 200 μm. **J** dCas9 CRISPR-FTO conjugates with sgRNA targeting A984 adjacent area of IGFL2-AS1 also increased the stability of IGFL2-AS1. 5 μM ActD was used. **K** dCas9 CRISPR-FTO conjugates with sgRNA targeting A984 adjacent area of IGFL2-AS1 dissociated it from YTHDF2 binding. *P < 0.05; **P < 0.01; **P < 0.001.

The aforementioned results pointed the up-regulation of IGFL2-AS1 in pazopanib resistant cells. However, H3K4me3 marks ahead of *IGFL2-AS1* between pazopanib sensitive and resistant cells were comparably viewed, indicating IGFL2-AS1 induction by chronic pazopanib treatment was not caused by transcriptional regulation (Supplementary Fig. 4A-B). Further investigation revealed that IGFL2-AS1 was more stable in pazopanib resistant ccRCC cells than that in sensitive cells (Supplementary Fig. 4C). Since m^6^A modification on RNA can affect its stability, we first examined whether IGFL2-AS1 was a m^6^A modified lncRNA. Analysis on miCLIP dataset (GSE161304) revealed that several m^6^A peaks were identified in *IGFL2-AS1* gene locus (Fig. 5A), suggesting IGFL2-AS1 was a potential m^6^A modified lncRNA. SRAMP m^6^A predictor indicated three sites (A76, A201, A984) having very high confidence of m^6^A modification (Supplementary Fig. 4D & Fig. 5B). To confirm this, we precipitated m^6^A modified RNAs with m^6^A antibody and found IGFL2-AS1 fragments were noticeably enriched in both OSRC-2 and A498 cells, which were hardly precipitated with m^6^A antibody in pazopanib resistant controls (Fig. 5B), suggesting IGFL2-AS1 preferred to be m^6^A modified in pazopanib sensitive ccRCC cells and these modifications were lost upon chronic pazopanib treatment. In addition, we noted a decreased expression of METTL14 in METTL3/METTL14 complex but not an increase of demethylase FTO or ALK in pazopanib resistant ccRCC cells (Fig. 5C), implying METTL14 reduction may account for IGFL2-AS1 demethylation. Indeed, METTL14 inhibition with two dependent siRNAs evidently reduced the m^6^A levels of IGFL2-AS1 (Fig. 5D), leading to ts expression (Fig. 5E). The expression level of IGFL2-AS1 was also negatively correlated with METTL14 expression in TCGA-KIRC dataset (Fig. 5F).

To examine which site was responsible for IGFL2-AS1 stability, we depleted m^6^A modification via dCas9 CRISPR-FTO site directed mutagenesis technique. As shown in Fig. 5G, A984 site directed demethylation significantly increased IGFL2-AS1 level in both A498 and OSRC-2 cells. Along with this, expression levels of AR/TWIST1, VM development and IGFL2-AS1 stability in both A498 and OSRC-2 were also increased by this site-directed demethylation (Fig. 5H–J), indicating the removal of m^6^A at A984 of IGFL2-AS1 was indispensable for its increase and the associated biological functions.

Early studies have reported that YTHDF2 recognizes some m^6^A RNAs and destabilizes them [28, 29]. Therefore, we hypothesized that m^6^A modified IGFL2-AS1 was recognized and destabilized by YTHDF2, which was reversed upon chronic pazopanib treatment. To test this speculation, we performed YTHDF2 RIP and found the interaction of IGFL2-AS1 with YTHDF2 was significantly decreased in pazopanib-resistant cells as compared to sensitive controls (Fig. 5K).

Taking together, we concluded that IGFL2-AS1 is m^6^A modified lncRNA, which is demethylated by METTL3/METTL14 and stabilized by YTHDF2 dissociation to promote AR expression upon pazopanib treatment.

### Assessment of the clinical values of IGFL2-AS1 in ccRCC patients

**Fig. 6:**
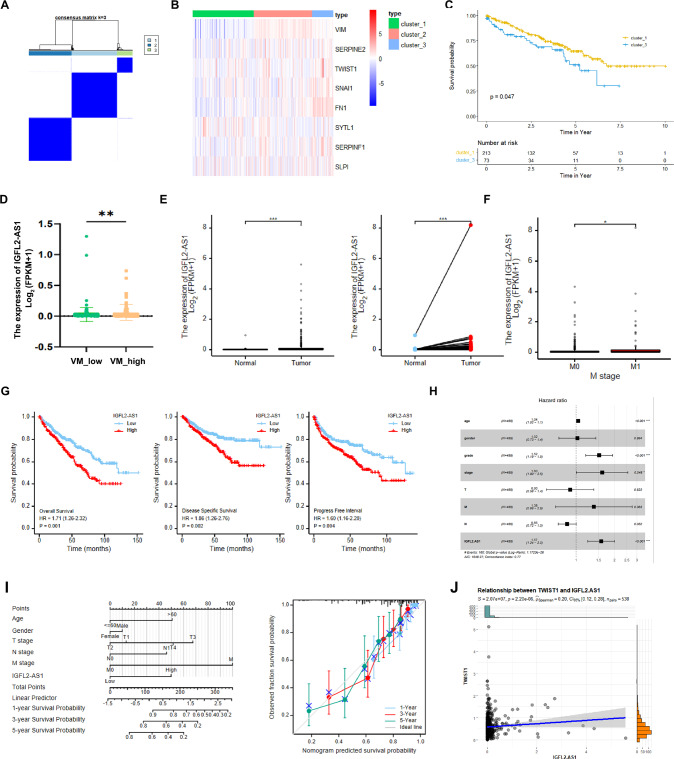
Assessment of the clinical values of IGFL2-AS1 in ccRCC patients. **A** Consensus matrix when *k* = 3. **B** A heat-map of expression pattern of VM related genes in individual cluster. **C** VM_high (cluster 3) patients experienced shorter survival time than VM_low (cluster 1) counterparts. **D** IGFL2-AS1 was over-expressed in VM_high subtype ccRCC. **E** the expression level of IGFL2-AS1 was evidently higher in ccRCC as compared to normal kidney tissues. **F** IGFL2-AS1 was highly expressed in distant metastatic ccRCC as compared to localized controls. **G** Kaplan–Meier survival analyses indicated ccRCC patients with high IGFL2-AS1 levels had worse OS (Overall Survival), DSS (Disease Free Survival) and PFI (Progression Free Interval) than those with low IGFL2-AS1 level. **H** Multivariate Cox regression analysis of Age, Gender, TNM, T stage, grade and IGFL2-AS1. **I** IGFL2-AS1 based Nomogram prediction model. **J** IGFL2-AS1 and VM gene TWIST1 was highly correlated at expression level in TCGA-KIRC. *P < 0.05; **P < 0.01; ***P < 0.001.

To assess the clinical value of IGFL2-AS1 in ccRCC patients, we first performed consensus clustering analysis by inputting VM related genes. CDF curve indicated *k* = 3 as the best category number (Fig. 6A & Supplementary Fig. 5A), which was clearly distinguished according to the PCA and TSN analyses (Supplementary Fig. 5B, C). We noticed VM related markers were more abundantly enriched in cluster 3 as compared to cluster 1 and cluster 2 (Fig. 6B). For this reason, we referred cluster 3 as VM_high ccRCC subtype and cluster 1 as VM_low ccRCC subtype. Survival analysis indicated that VM_high cluster 3 patients experienced shorter overall survival rate as compared to VM_low cluster 1 cohorts (*p* = 0.047, Fig. 6C). Consistently, a significant increase of IGFL2-AS1 level was observed in VM_high cluster 1 patients (Fig. 6D). As long with this, high level of IGFL2-AS1 was also positively correlated with ccRCC tumor initiation, distant metastasis, pathological stage and histologic grade (Fig. 6E, F & Supplementary Fig. 5D). ccRCC patients with high IGFL2-AS1 expression had worse OS (Overall Survival), DSS (Disease Specific Survival) and PFS (Progression Free Survival) than the corresponding counterparts (Fig. 6G).

Owing to the clinical significance of IGFL2-AS1 in ccRCC, we generated a IGFL2-AS1 based nomogram prediction model for clinically OS evaluation. As exhibited in Fig. 6I, eight independent prognostic variables including age, gender, TNM stage, pathological stage, histologic grade and IGFL2-AS1 expression were recorded and assigned with different points according to the multivariate Cox regression analysis (Fig. 6H). 1-,3-,5- year probabilities were gained by drawing the total point downward to the bottom 0-1 probability scales and subjected to fitness test. As the key factor determining VM development, the expression level of IGFL2-AS1 was also positively correlated with the expression level of AR downstream effector TWIST1 in TCGA-KIRC (Fig. 6J).

Together, results from Fig. 6A–J confirm that IGFL2-AS1 is clinically correlated with VM formation and progression.

### Preclinically targeting AR with enzalutamide or IGFL2-AS1 with siRNAs to suppress pazopanib resistant ccRCC progression

**Fig. 7:**
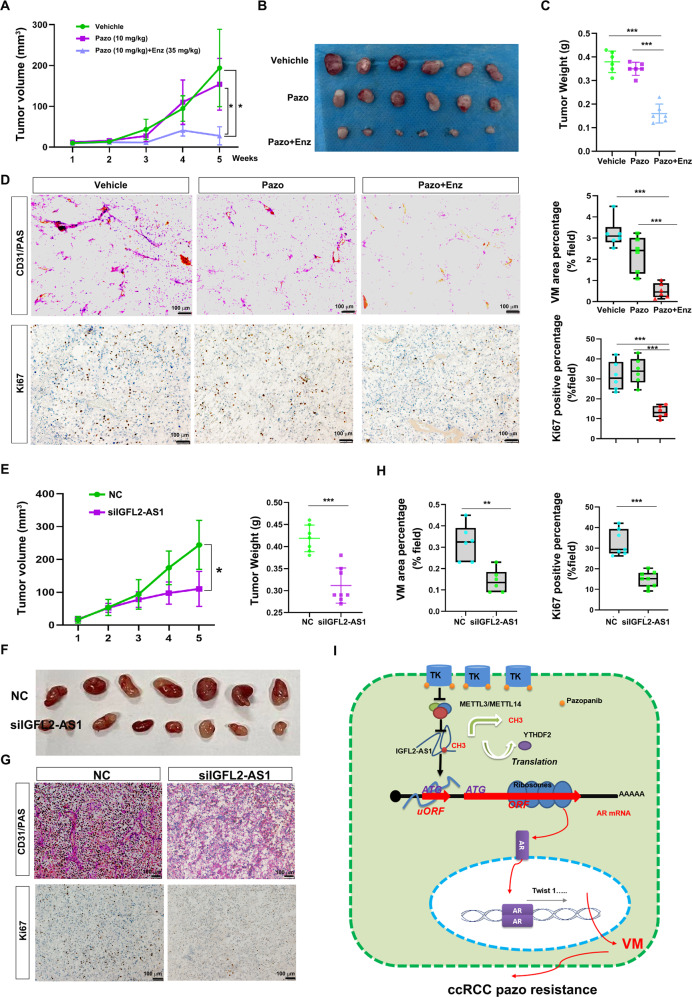
Preclinically targeting AR with enzalutamide or IGFL2-AS1 with siRNAs to suppress pazopanib resistant ccRCC progression. **A**. Tumor growth curve of pazopanib resistant OSRC-2 tumors which received indicated therapies. 10 mg/kg pazopanib and 35 mg/kg enzalutamide were intraperitoneally injected every other day. **B** Tumor images from sacrificed mice in **A**. **C** Tumor weight from sacrificed mice in **A**. **D** CD31/PAS and Ki67 staining assays of indicated tumor sections. Left, representative images. Right, statistical analyses. Scale bar: 100 μm. **E** Tumor growth curve (left) and tumor weight (right) of pazopanib resistant OSRC-2 tumors which received siNC (8 mg/kg) or siIGFL2-AS1 (8 mg/kg) administration. **F** Tumor images from sacrificed mice which received siNC or siIGFL2-AS1 administration. **G** CD31/PAS and Ki67 staining assays of siNC or siIGFL2-AS1 treated tumor sections. Scale bar: 100 μm. **H** statistical analyses of CD31/PAS and Ki67 staining assays in **G**. **I** Schematic depiction of how m^6^A mediated IGFL2-AS1/AR axis controls VM development and pazopanib resistance in ccRCC. *P < 0.05; **P < 0.01; ***P < 0.001.

To verify our in vivo findings in the in vivo mouse model, we first subcutaneously implanted pazopanib-resistant OSRC-2 cells into male nude mice and developed RCC tumors. When tumors reached appropriate size, mice were divided into 3 groups (*n* = 6 for each group) and the following therapies were administrated: 1) vehicle; 2) 10 mg/kg pazopanib; 3) 10 mg/kg pazopanib plus 30 mg/kg enzalutamide. Data showed that pazopanib administration failed to suppress tumor growth (Fig. 7A, B), enforcing the drug-resistant property of this ccRCC tumor. However, combined administration of pazopanib and enzalutamide significantly suppressed ccRCC tumor growth, suggesting targeting AR with its inhibitor enzalutamide could enhance pazopanib efficacy (Fig. 7A, B). We then sacrificed mice and examined VM formation by staining CD31/PAS. As result, VM development was impaired by enzalutamide administration, along with this was the decreased cell proliferation rate monitored by Ki67 staining (Fig. 7C).

The contribution of IGFL2-AS1 to ccRCC progression was also validated by delivering in vivo siRNAs into pazopanib resistant OSRC-2 tumors (*n* = 7 for vehicle treated group, *n* = 8 for siRNAs treated group), which revealed that siRNAs targeting IGFL2-AS1 remarkably slowed down tumor growth as well as decreased VM number (Fig. 7D–F), strengthening the role of IGFL2-AS1 in ccRCC progression via altering its VM development.

Together, all results suggest that chronic pazopanib treatment reduces m^6^A level of lncRNA IGFL2-AS1, which is stabilized due to YTHDF2 release and binds the 5’-UTR of AR mRNA, inhibiting its uORF activity and favoring its translation. Finally, the activation of IGFL2-AS1/AR axis leads to pazopanib resistance via facilitating ccRCC VM development (Fig. 7G).

## Discussion

Metastatic ccRCC patients have poor prognosis but benefit from current standardized treatments such as immunotherapy or immunotherapy plus TKI. However, TKI monotherapy is still an alternative option for ccRCC patients who cannot tolerate immunotherapy. Moreover, immunotherapy may cause a great economic burden to tumor bearing patients. All these facts lead some developing countries to continuously apply TKI such as pazopanib as the first-line therapy to treat metastatic ccRCC, which also successfully prolongs patients’ survival. However, its durability is not sustained for metastatic ccRCC with only a limited therapeutic duration for estimated 12 months [30]. One potential mechanism responsible for the loss of therapy efficacy is the ccRCC tumor resistance to pazopanib, such as alternative means to establish its vasculature. As tumor derived vessel like vascular networks, VM was identified by us as one alternative way to provide blood or nutrients for pazopanib resistant ccRCC tumor when the angiogenesis was inhibited. Mechanistic explorations suggested that the activation of IGFL2-AS1/AR signaling was at least partially responsible for pazopanib induced VM formation and the subsequent drug resistance. IGFL2-AS1 was demethylated at several m^6^A sites by METTL3/METTL14 complex and stabilized by the dissociation of YTHDF2. Stabilized IGFL2-AS1 bond the 5’-UTR of AR mRNA and inhibited its activity, favoring AR translation. Finally, the in vivo xenografted mouse model also suggested that targeting IGFL2-AS1 with siRNA or AR with enzalutamide suppressed pazopanib resistant ccRCC progression. Overall, our study identifies IGFL2-AS1 mediated AR signaling as the key to bridge VM formation and pazopanib resistance and targeting this signaling axis could overcome resistance of ccRCC cells to pazopanib treatment.

Male dominant ccRCC implies the possible involvement of sex hormone receptor AR in the development of this disease. Although early studies have emphasized the significant role of AR in ccRCC tumour growth, tumor metastasis and angiogenesis [22, 25–27], no evidence link its contribution to pazopanib resistance. Our results illustrated that pazopanib induced AR expression caused drug resistance by increasing VM number. Further mechanistic dissection showed that pazopanib increased AR level was controlled by IGFL2-AS1, which directly regulated its upstream open reading frame (uORF) in the 5’-UTR region. An early literature has documented that uORFs enable their downstream proteins to be 30–80% decreasingly expressed, having serious impacts on various biological events including cancer development [31]. Consistent to this report, IGFL2-AS1 negatively influenced uORF translation via direct contact, thus enhancing the translation of AR coding sequence. To the best of our knowledge, this study is the first one providing experimental evidence to support the negative regulation of uORF on its downstream coding sequence. Also, our study enriches the regulatory networks around AR in ccRCC, helping scientists better understand the role of AR in this disease.

The non-coding RNAs (lncRNAs) are molecules with length more than 200 base pairs, which are originally considered as non-functional “trash” [32, 33]. However, accumulating evidence illustrate that lncRNAs are functionally involved in both physiological and pathological processes via a variety of means [33]. Herein, lncRNA IGFL2-AS1 interacts with the AR mRNA to exert its cancer promoting function. Generally speaking, lncRNA interacts with targeted mRNA to alter its stability via recruiting RNA binding protein (RBP). For example, lncRNA LAST recruits RBP protein CNBP to the 5’-UTR of its targeted CCND1 mRNA in order to protect it from nuclease cleavage [34]. Another case demonstrates that lncRNA Sros1 interacts with STAT1 mRNA and prevents it from binding to RBP protein CAPRIN1, increasing STAT1 stability [35]. Unusually, lncRNA IGFL2-AS1 binds to AR mRNA and blocks the potential translation activity of its uORF, thus favoring the translation of downstream AR ORF. Therefore, this study refreshes current knowledge of means by which lncRNA influences the expression of its targets.

Amounting evidence suggest that post-transcriptional modification on RNA, also called epitranscriptomic modification, plays critical role in gene regulation [36]. N6-methyladenosine (m^6^A), tightly controlled by writers (METTL3/METTL14 complex), erasers (FTO, ALKBH5) and readers (YTH domain-containing proteins), is the most common modification on RNA, affecting its splicing, stability and translation [37]. Herein, our results revealed that IGFL2-AS1 was m^6^A modified and this modification was reversibly removed upon chronic pazopanib treatment. Early studies have demonstrated that reader YTHDF2 can recognize and destabilize m^6^A modified RNAs and dragging them to the decaying cellular machinery [28]. Consistently, demethylated IGFL2-AS1 at m^6^A sites weakened its binding with YTHDF2, being more stable and highly expressed in pazopanib-resistant cells to promote AR expression and the subsequent VM formation. However, how a decreased global m^6^A level is caused in pazopanib-resistant ccRCC cells is still open to discuss and deserves intensive investigations.

Together, our study has proved that VM formation is an essential route for ccRCC cells to acquire pazopanib resistance, which is probably mediated by VM related genes. During this process, IGFL2-AS1 is demethylated and stabilized to interact with the 5’-UTR of AR mRNA, facilitating its translation. Targeting either IGFL2-AS1 or AR may be an alternative strategy to increase the efficacy of pazopanib.

## Material and Methods

### Cell culture

STR authenticated A498, OSRC-2, 293 T cell lines were purchased from IMMOCELL Biotech CO.,LTD (Xiamen, Fujian, China). Cells were maintained in DMEM containing 10% certified Fetal Bovine Serum FBS (VivaCell, Shanghai, China), 2 mM L-glutamine, 100 IU/ml penicillin and 100 ug/ml streptomycin at 37 °C in humidified 5% CO_2_ environment.

### Plasmids construction

Wild type or mutated IGFL2-AS1 sequence was synthesized and constructed into pWPI backbone digested with Pac I and Pme I restriction enzymes. shRNAs were annealed and ligated to PLKO backbone digested with Age I and EcoR I restriction enzymes.

### Lentivirus packaging

Lenti-plasmids (PLKO, pWPI) with interested DNA sequences were co-transfected with psPAX2 and pMD2G into 293 T cells using the standard calcium chloride transfection method. 48 h later, lentivirus supernatant was collected and concentrated by sucrose gradient centrifugation, then incubated with ccRCC cells or frozen for future use.

### PAS/CD31 staining

Deparaffinized ccRCC sections were stained with CD31 as described in immunohistochemical staining protocol and followed by PAS staining (ab150680). Briefly, sections were incubated with Periodic Acid reagent at RT temperature for 10 min and followed by 4 times water wash, then incubated with Schiff’s Solution at RT temperature for 15 min and stained nuclei with hematoxylin.

### Vascular Mimicry (VM) formation assay and quantification

A498 and OSRC-2 cells with or without gene manipulation were trypsinized and suspended in 10% FBS DMEM at ~2 × 10^5^/ml cell concentration. Growth factor-reduced matrigel (Corning Inc., USA) was plated to 96-well plates at a horizontal level. Then 100 μl of re-suspended ccRCC cells were cultured on top of matrigel at 37 °C for 6 h, each well was analyzed under a microscope. VM quantification was made by calculating the branching points: branching points are created by either a new vessel sprout (branching) or two separate vessels fusing into one. Angiogenesis Analyzer Plugin in Image J software was used to calculate the branching points of three independent VM images.

### Transwell invasion assay

Standard matrigel (356235, Corning) was 1:5 diluted with serum-free DMEM medium and seeded to 8 µm pore sized upper chamber (Corning, Inc., USA). ccRCC cells with gene manipulation were trypsinized and seeded into the upper chambers at 5 × 10^4^ cells/well concentration. and 10% FBS DMEM medium was added to the bottom chambers as attractant. After 16 h, the invaded cells were fixed by 75% ethanol and stained with 0.1% crystal violet. Statistical analysis of invaded cells was made in image J software and normalized to the proliferative index of indicated cells.

### RNA immunoprecipitation

The pazopanib sensitive and resistant ccRCC cells were lysed in DEPC lysis buffer (with RNase inhibitor) for 30 min. The cell suspension was centrifuged at speed of 14,000 prm for another 10 min. The supernatants were incubated with biotin labeled 5 nM AR anti-sense probes overnight at 4 °C. AR mRNA complex was pulled down with 10 µl Streptavidin Agarose beads. After intensive washes, RNAs in the beads were extracted using Trizol reagent and subjected to RT-PCR analysis RT-QPCR to detect IGFL2-AS1. AR anti-sense probes were listed in Supplementary Table 1.

### In Situ hybridization (FISH)

A498 and OSRC-2 pazopanib resistant cells were fixed by 4% paraformaldehyde and permeabilized in 0.3% Triton X-100 PBS, then hybridized with the two biotin-labeled probes (5 nM) at 50 °C overnight. Cells were then intensively washed and incubated with streptavidin Alexa Fluor 488-conjugated IgG (S11223, ThermoFisher) at 4 °C overnight. DAPI was utilized to stain nuclei. Fluorescent images were captured by confocal microscope.

### Cyto-nuclear fraction separation

Cells were trypsinized and washed with cold PBS two times. Cells were suspended in Buffer A (10 mM HEPES pH 7.9, 1.5 mM MgCl_2_, 10 mM KCl, 1 mM DTT) on ice for 15 min and centrifuged at speed of 14,000 prm for 10 min. The top supernatant was cytoplasmic fraction while the bottom pellets referred as nuclear fraction. These fractions were subjected to cytoplasmic and nuclear RNAs extraction using Trizol reagent (TIANGEN BIOTECH CO.,LTD, Beijing).

### RNA extraction and qRT-PCR

Total RNAs were isolated from pazopanib-resistant and sensitive OSRC-2 and A498 cells using Trizol reagent. 2 µg RNA was used as a template for the reverse transcription with ReverTra Ace™ qPCR RT Kit (TOYOBO, Japan). Quantitative real-time PCR (qRT-PCR) was conducted in LightCycler 480 with SYBR Green dye (TOYOBO, Japan) to determine the mRNA expression level of a gene of interest. Calculation of relative gene expression was performed using the 2^-^^^CT method. Expression levels were normalized to the expression of GAPDH mRNA. Primers used in this study were listed in Supplementary Table 2.

### Western blot analysis

Cells were lysed using lysis buffer (50 mM Tris-HCl/pH 7.4, 1% NP-40, 150 mM NaCl, 1 mM EDTA, 1 mM PMSF, 1 mM Na3VO4, 1 mM NaF) containing a protease inhibitor cocktail. Samples containing 20–40 μg of total proteins were separated by 10–15% sodium dodecyl sulfate (SDS)-PAGE and transferred onto a polyvinylidenedifluoride membrane. After the block of the membranes with 5% fat free milk in TBST (50 mM Tris/pH 7.5, containing 150 mM NaCl and 0.05%Tween-20) for 1 h at room temperature, the membranes were incubated with appropriate dilutions of specific primary antibodies overnight at 4 °C, followed by HRP-conjugated secondary antibodies and visualized using ECL system (Thermo Fisher Scientific Inc). AR (N-20, Santa Cruz), GAPDH (2118, CST) and TWIST1 (46702, CST) antibodies were utilized in this study.

### Luciferase reporter assay

The 5’-UTR of AR mRNA with or without IGFL2-AS1 binding region was constructed into pGL3-basic vector. Stable ccRCC cells expressing wild type and mutated IGFL2-AS1 were seeded in 24-well plates and pGL3 constructs (200 ng/well) were co-transfected with pRL-TK (10 ng/well) using lipofectamine 3000 (Invitrogen) according to the manufacturer’s instruction. 48 h later, luciferase activity was measured by Dual-Luciferase Assay Kit (Promega).

### Consensus clustering analysis

TCGA-KIRC patients were classified into VM_high and VM_low subtypes by “ConsensusClusterPlus” R package using VM related genes as inputs. The data was visualized in two dimensions by Principal component analysis (PCA) and t-distributed stochastic neighbor embedding (t-SNE) analysis with “ggplot2” and “Rtsne” package, respectively. The expression patterns of VM related genes and IGFL2-AS1 were visualized by “pheatmap” R package.

### Immunohistochemical staining (IHC)

The deparaffinized ccRCC sections (informed consent was obtained from patients and experiments were performed with the approval of the Ethics Committee of the Affiliated Hospital of Southwest Medical University) or cell line derived tumor sections were treated with methanol containing 3% peroxidase for 15 min and followed by antigen retrieval in citrate buffer (pH = 6.0) at 98 °C for 10 min. Sections were then blocked by 5% BSA + 5% milk PBS for 1 h at room temperature and followed by incubation with anti-AR (1:100, N-20, Santa Cruz) or Ki-67 (ab15580, abcam) at 4 °C overnight. Then biotin-labeled secondary antibody was added for another 30 min, followed by 30 min streptavidin incubation (PK-4000, Vectastain, Burlingame, CA.) and DAB (SK-4100, Vectastain, Burlingame, CA) was used to determine AR or Ki-67 signals. Image J software was used to determine AR IHC score.

### In vivo studies

1 × 10^6^ ORSC-2 cells suspended in DMEM medium were mixed with matrigel (1:1, v/v) and subcutaneously implanted to the skin of 6–8 weeks male nude mice, which will receive various therapies as described in the results section: negative siRNA (8 mg/kg); siIGFL2-AS1 (8 mg/kg); enzalutamide (35 mg/kg). Invivofectamine 2.0 kit (#1377501, Invitrogen) was used to deliver the siRNA as previously described. Tumors were measured individually by caliper. Finally, mice were sacrificed and tumors were removed for CD31/PSA staining. Animal experiments were performed under the supervision of the animal committee of Southwest Medical University. G*power software was used to calculate the proposed n number of animal size (the presented n number may vary due to multiple reasons during animal experiment).

### Statistical analysis

All values are reported as the mean ± SD and all comparisons were analyzed with one way ANOVA followed by *t*-test in Graphpad Prism 8.0. Chi-square test was used to analyze the significant difference of between AR IHC score with VM number. *P* < 0.05 was viewed as statistically significant.

## Supplementary information

Sfig 1

Sfig 2

Sfig 3

Sfig 4

Sfig 5

Original Data File

supplementary Figure legend

Supplementary Table 1

Supplementary Table 2

## Data Availability

The raw data and materials related to this study will be provided if there is a reasonable request.
