# Supplementary figures and images for: Vascular mimicry induced by m6A mediated IGFL2-AS1/AR axis contributes to pazopanib resistance in clear cell renal cell carcinoma
Source: Cell Death Discov. 2023 Apr 11;9:121. doi: 10.1038/s41420-023-01423-z (PMC10086028; doi:10.1038/s41420-023-01423-z)

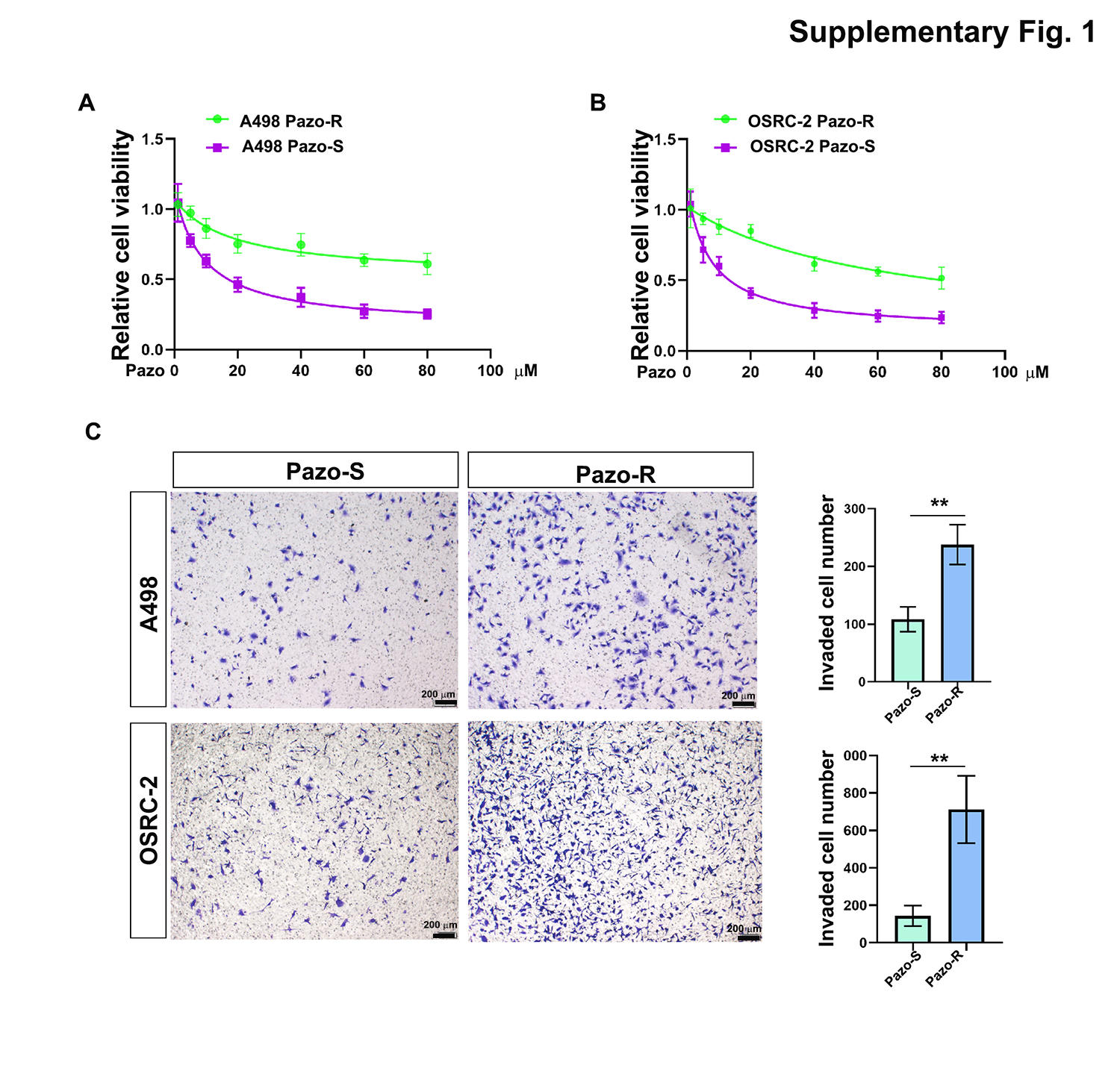

Supplement: Supplementary file 1 — Sfig 1 [file 41420_2023_1423_MOESM1_ESM.tif]

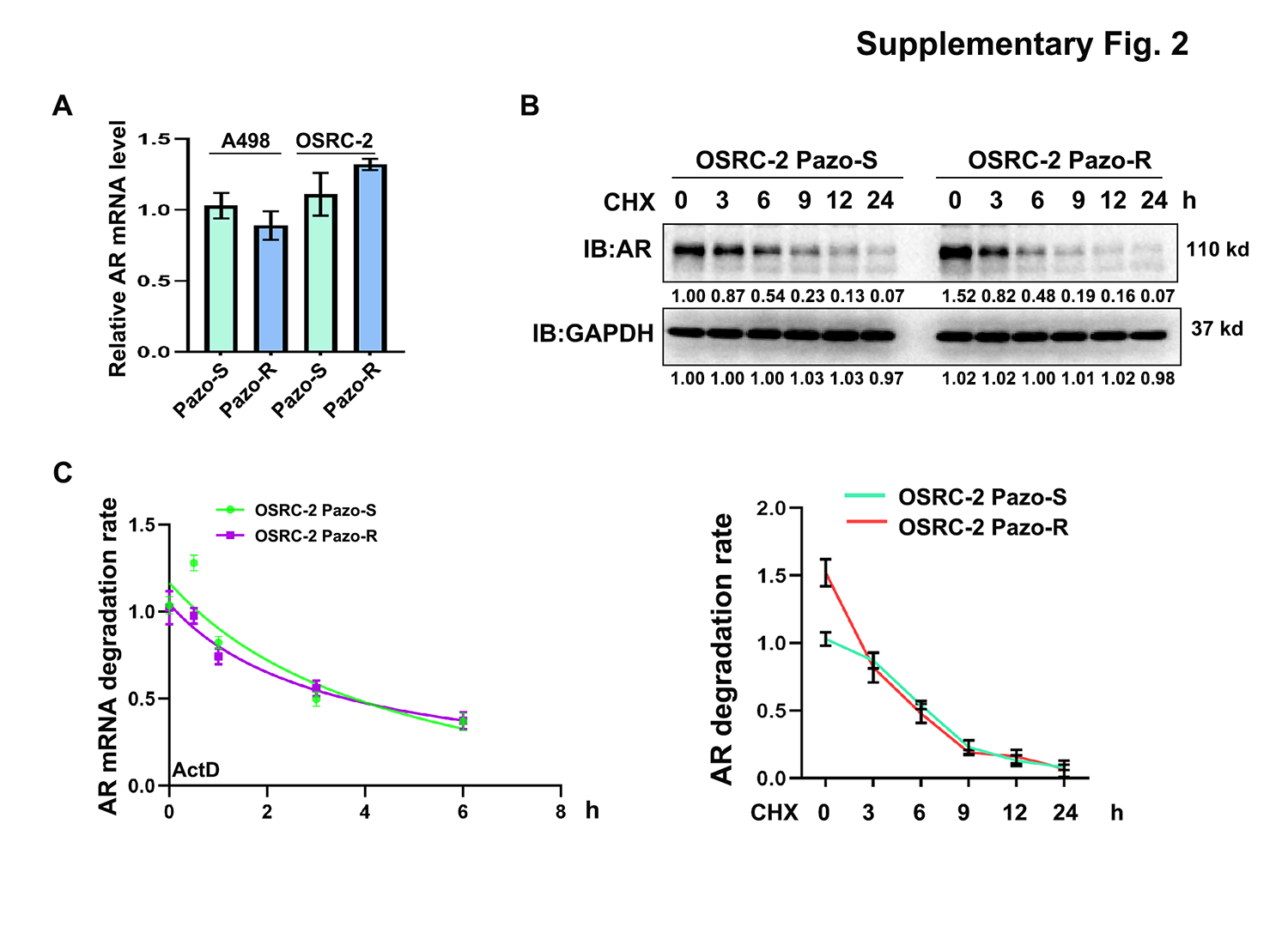

Supplement: Supplementary file 2 — Sfig 2 [file 41420_2023_1423_MOESM2_ESM.tif]

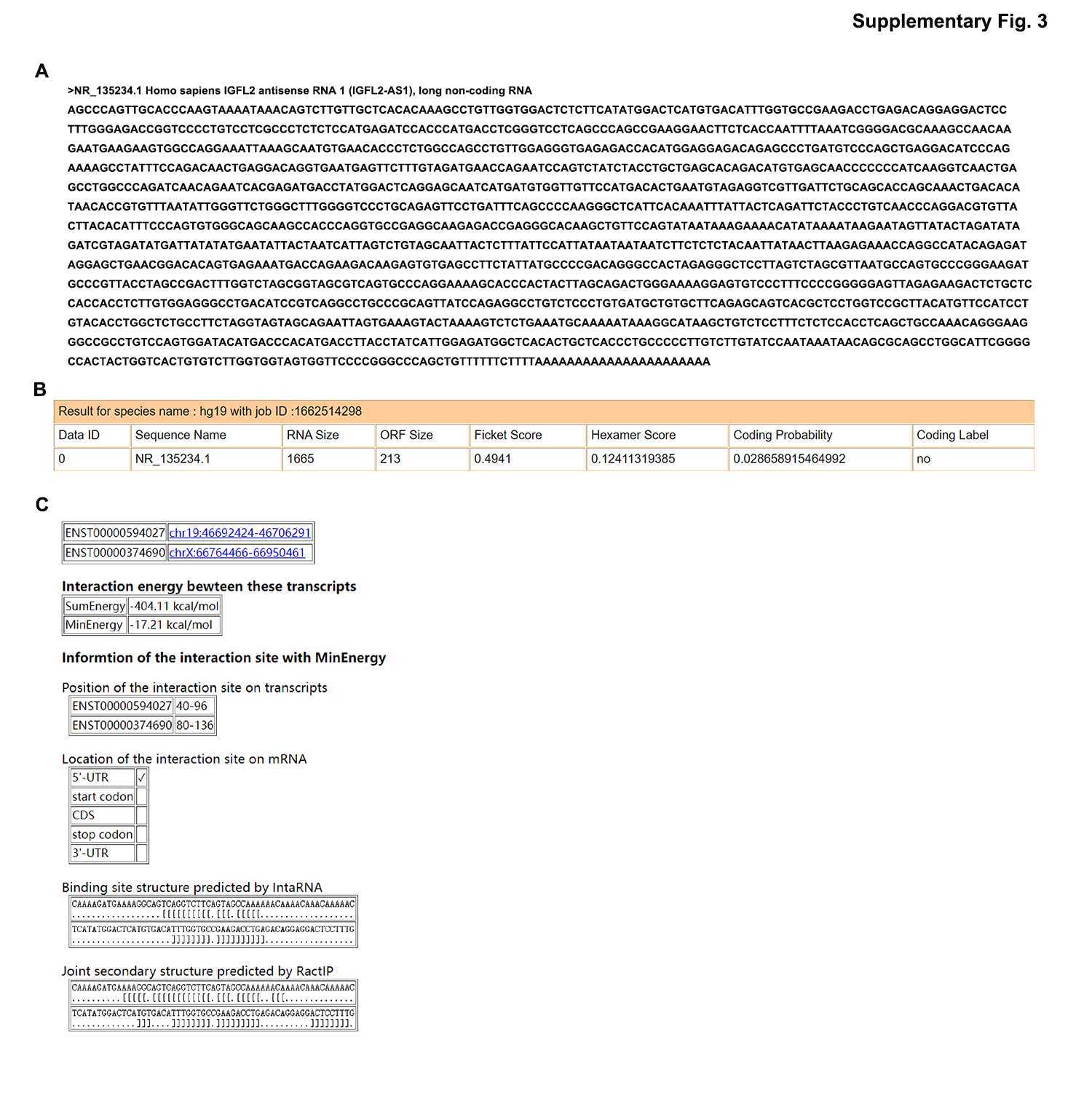

Supplement: Supplementary file 3 — Sfig 3 [file 41420_2023_1423_MOESM3_ESM.tif]

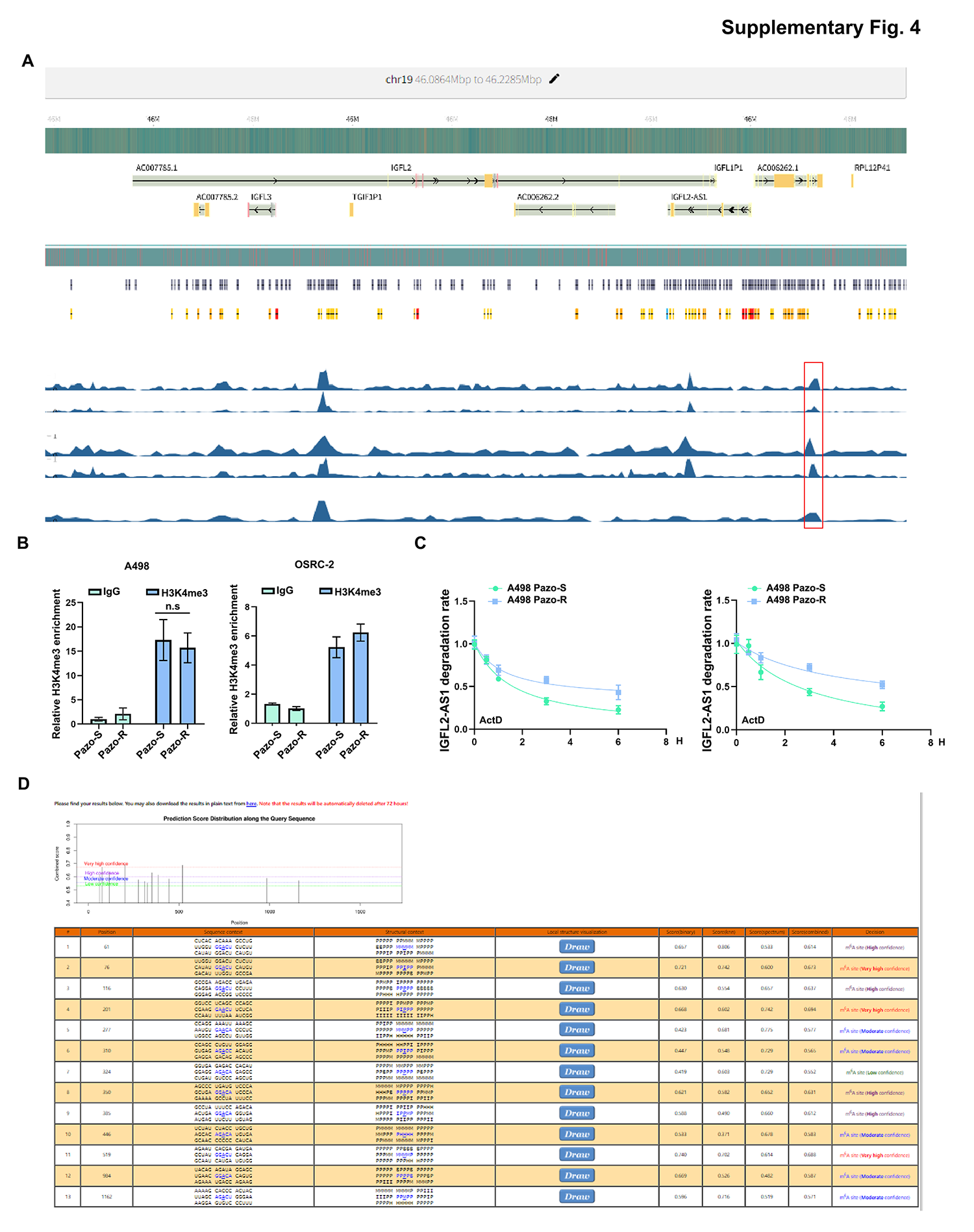

Supplement: Supplementary file 4 — Sfig 4 [file 41420_2023_1423_MOESM4_ESM.tif]

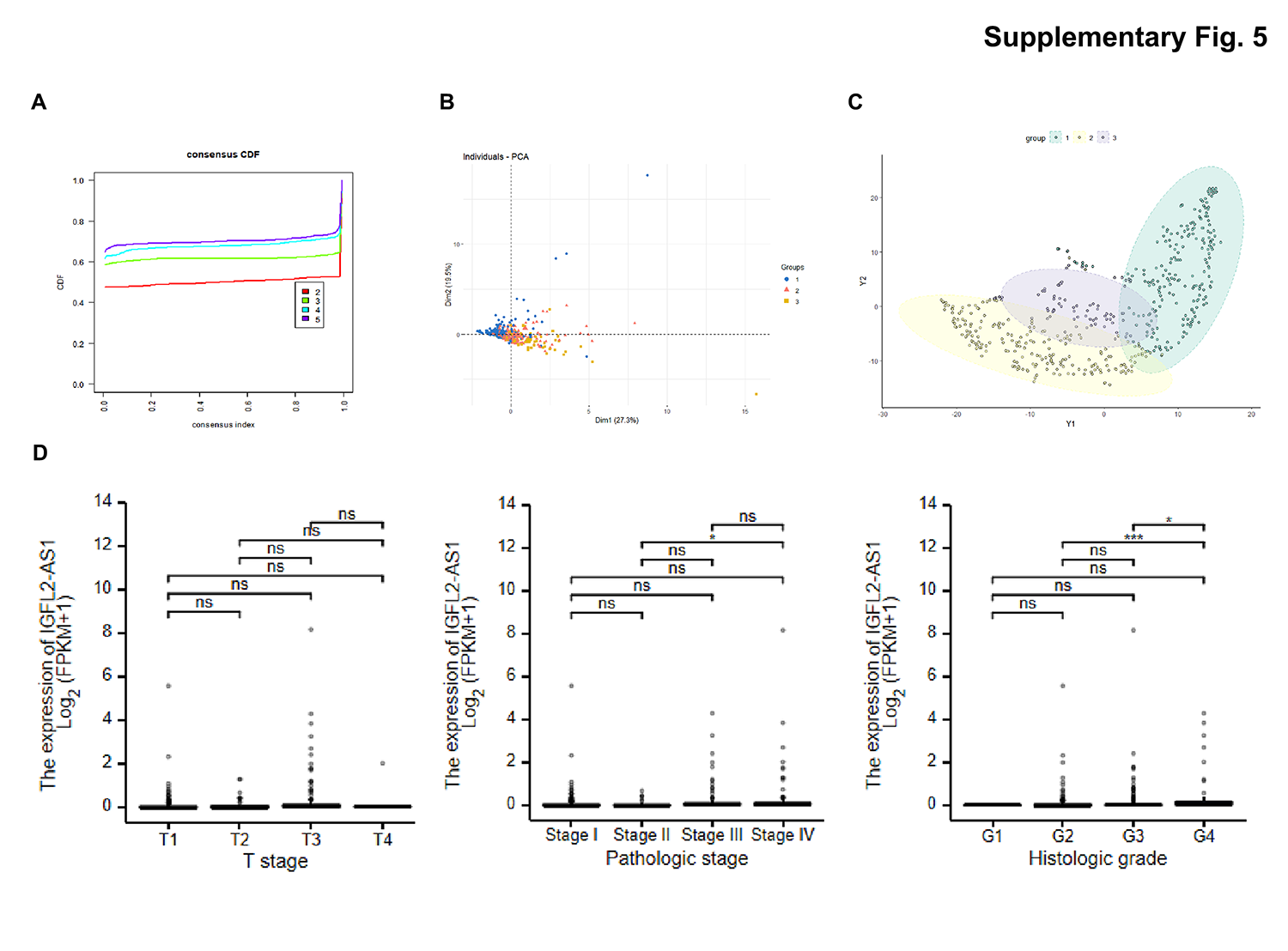

Supplement: Supplementary file 5 — Sfig 5 [file 41420_2023_1423_MOESM5_ESM.tif]

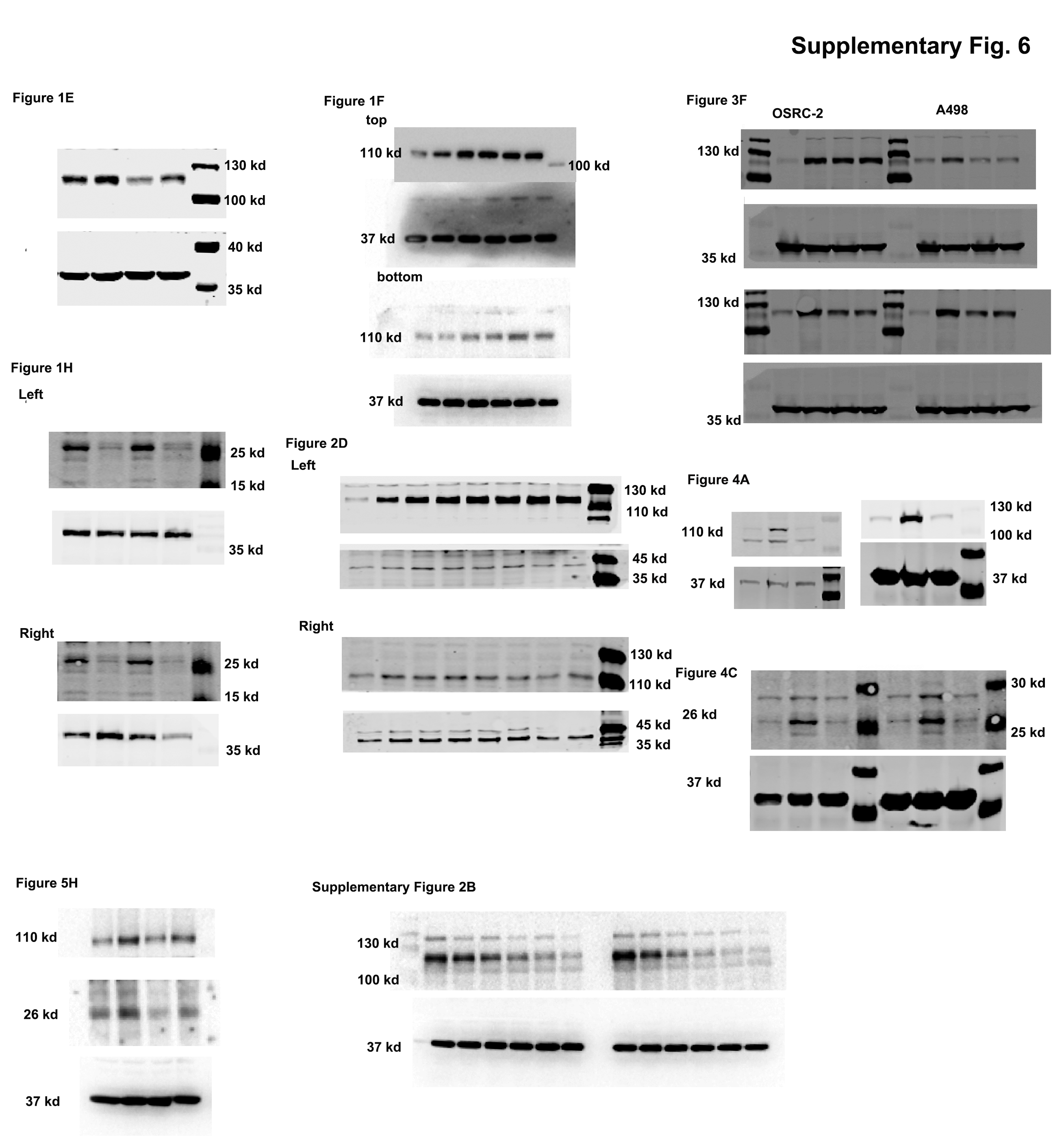

Supplement: Supplementary file 6 — Original Data File [file 41420_2023_1423_MOESM6_ESM.tif]
