# Supplementary material for: Vascular mimicry induced by m6A mediated IGFL2-AS1/AR axis contributes to pazopanib resistance in clear cell renal cell carcinoma
Source: Cell Death Discov. 2023 Apr 11;9:121. doi: 10.1038/s41420-023-01423-z (PMC10086028; doi:10.1038/s41420-023-01423-z)
Supplement: Supplementary file 7 — supplementary Figure legend [file 41420_2023_1423_MOESM7_ESM.doc]

**Supplementary Figure legend**

**Figure 1. A.** Pazopanib sensitivity test of A498 pazopanib resistant and sensitive cells.  **B.** Pazopanib sensitivity test of OSRC-2 pazopanib resistant and sensitive cells. **C.** OSRC-2/A498 pazopanib resistant cells had better capability to invade than sensitive controls. Left, representative images of invaded cells. Right, statistical analyses. Scale bar: 100 μm. *P**<0.05; *P***<0.01.

**Figure 2. A.** The mRNA level of AR in pazopanib resistant and sensitive cells. Gene expression was normalized to GAPDH mRNA. **B**. AR protein stability comparison of OSRC-2 pazopanib resistant and sensitive cells. 10 μM CHX was used to inhibit protein synthesis. GAPDH was loading control. **C**. AR mRNA stability in OSRC pazopanib resistant and sensitive cells. 5 μM ActD was used to inhibit mRNA synthesis. *P**<0.05; *P***<0.01.

**Figure 3. A.** IGFL2-AS1 sequence**. B,** Coding Potential Assessment Tool shows the coding probability of IGFL2-AS1. **C.** The interaction of IGFL2-AS1 with AR mRNA predicted by IntaRNA and RactIP.

**Figure 4.** **A**. Encode Project showing the H3K4me3 peaks upstream of *IGFL2-AS1* locus. **B.** IGFL2-AS1 was more stable in pazopanib resistant cells than sensitive controls. **C**. SRAMP m^6^A prediction of IGFL2-AS1 *P**<0.05; *P***<0.01.

**Figure 5**. **A**. The CDF curve of consensus clustering analysis using VM related genes. **B-C**. PCA (B) and TSN (C) analysis of consensus clustering analysis using VM related genes. **D**. IGFL2-AS1 expression pattern in different T stage, pathological stage and histologic grade of TCGA-KIRC dataset. *P**<0.05; *P****<0.001, n.s=no significance.
