# Supplementary material for: Vascular mimicry induced by m6A mediated IGFL2-AS1/AR axis contributes to pazopanib resistance in clear cell renal cell carcinoma
Source: Cell Death Discov. 2023 Apr 11;9:121. doi: 10.1038/s41420-023-01423-z (PMC10086028; doi:10.1038/s41420-023-01423-z)
Supplement: Supplementary file 8 — Supplementary Table 1 [file 41420_2023_1423_MOESM8_ESM.docx]

| **Primer Name** | **Sequence (5’-3’)** |
| --- | --- |
| shIGFL2-AS1 F  shIGFL2-AS1 R  shAR F  shAR R  sgRNA(76)  sgRNA(201)  sgRNA(984)  AR probe#1  AR probe#2  AR probe#3  AR probe#4  IGFL2-AS1 probe#1  IGFL2-AS1 probe#2 | CCGGGTGGCCAGGAAATTAAAGCAAGGATCCTTGCTTTAATTTCCTGGCCACTTTTTG  AATTCAAAAAGTGGCCAGGAAATTAAAGCAAGGATCCTTGCTTTAATTTCCTGGCCAC  CCGGCAGGCAAGAGCACTGAAGATAGGATCCTATCTTCAGTGCTCTTGCCTGTTTTTG  AATTCAAAAACAGGCAAGAGCACTGAAGATAGGATCCTATCTTCAGTGCTCTTGCCTG  CCAACAGGCTTTGTGTGAGCAA  GAGGUCAUGGGUGGAUCUCAUG  AUCUCUGUAUGGCCUGGUUUC  UCACUUCGCGCACGCUCUGGAACAGAUUCUGGAAAGCUCC-biotin  CGCUGUCGUCUAGCAGAGAACCUUUGCAUUCGGCCAAU-biotin  CACACUACACCUGGCUCAAUGGCUUCCAGGACAUUCAGAA-biotin  CAGCCCAUCCACUGGAAUAAUGCUGAAGAGUAGCAGUGCT-biotin  CUUCGGCACCAAAUGUCACAUGAGUCCAUAUGAAGAGAGU-biotin  CAGUGACCAGUAGUGGCCCCGAAUGCCAGGCUGCGCUGUU-biotin |

**Supplementary Table 1. Sequences of shRNAs, siRNAs and sgRNAs**
