# Supplementary material for: Vascular mimicry induced by m6A mediated IGFL2-AS1/AR axis contributes to pazopanib resistance in clear cell renal cell carcinoma
Source: Cell Death Discov. 2023 Apr 11;9:121. doi: 10.1038/s41420-023-01423-z (PMC10086028; doi:10.1038/s41420-023-01423-z)
Supplement: Supplementary file 9 — Supplementary Table 2 [file 41420_2023_1423_MOESM9_ESM.docx]

| **Primer Name** | **Sequence (5’-3’)** |
| --- | --- |
| AR-forward  AR-reverse  IGFL2-AS1-forward  IGFL2-AS1-reverse  METTL3-forward  METTL3-reverse  METTL14-forward  METTL14-reverse  YTHDF1-forward  YTHDF1-reverse  YTHDF2-forward  YTHDF2-reverse  YTHDF3-forward  YTHDF3-reverse  FTO-forward  FTO-reverse  ALKBH5-forward  ALKBH5-reverse  YTHDC1-forward  YTHDC1-reverse  YTHDC2-forward  YTHDC2-reverse  IGF2BP2-forward  IGF2BP2-reverse  IGF2BP3-forward  IGF2BP3-reverse | AATCCCACATCCTGCTCAAG  AAGTCCACGCTCACCATG  AGAAGACTCTGCTCCACCAC  CAGAGCCAGGTGTACAGGAT  TTGTCTCCAACCTTCCGTAGT  CCAGATCAGAGAGGTGGTGTAG  GAACACAGAGCTTAAATCCCCA  TGTCAGCTAAACCTACATCCCTG  ACCTGTCCAGCTATTACCCG  TGGTGAGGTATGGAATCGGAG  CCTTAGGTGGAGCCATGATTG  TCTGTGCTACCCAACTTCAGT  TCAGAGTAACAGCTATCCACCA  GGTTGTCAGATATGGCATAGGCT  GCTGCTTATTTCGGGACCTG  AGCCTGGATTACCAATGAGGA  CGGCGAAGGCTACACTTACG  CCACCAGCTTTTGGATCACCA  GAGGGCCAAATCTCCTACGC  GTCTCATGGTCAGAGCCATATTC  CAAAACATGCTGTTAGGAGCCT  CCACTTGTCTTGCTCATTTCCC  AGTGGAATTGCATGGGAAAATCA  CAACGGCGGTTTCTGTGTC  TATATCGGAAACCTCAGCGAGA  GGACCGAGTGCTCAACTTCT |

**Supplementary Table 2. Primers used in Real time PCR**
